# Changes and monitoring technology of human heart rate and blood oxygen saturation under high-altitude hypoxia

**DOI:** 10.3389/fphys.2025.1642777

**Published:** 2025-09-01

**Authors:** Yan Liao, Dianxiang Lu, Jin Yang

**Affiliations:** ^1^ College of Pharmacy, Chengdu University, Chengdu, China; ^2^ Clinical Medical College and Affiliated Hospital of Chengdu University, Chengdu, China

**Keywords:** high-altitude, hypoxia, heart rate, blood oxygen saturation, photoplethysmography, acute mountain sickness

## Abstract

High-altitude hypoxia affects human physiology and primarily regulates the cardiovascular system by hypoxia-inducible factor and relative factors. This review introduces physiological changes in heart rate and blood oxygen saturation, commonly used monitoring techniques, and their limitations for the diagnosis of acute mountain sickness (AMS). Under acute hypoxia, peripheral oxygen saturation (SpO_2_) decreases, and heart rate increases; under subacute hypoxia, SpO_2_ rebound but remain below sea level baseline values, and heart rate gradually decreases; under long-term hypoxia heart rate returns to baseline values at sea level, but SpO_2_ remains below them. Tibetans exhibit lower heart rate than Han Chinese at identical altitudes, while Andeans show elevated heart rate versus lowlanders. SpO_2_ reductions persist in Tibetans/Andeans but approach lowlander levels in Ethiopians. Cerebral oxygen saturation is also used as a complementary indicator of blood oxygen saturation and could be applied to the monitoring of high-altitude hypoxic level, but there are fewer studies in this area. Current heart rate and blood oxygen saturation monitoring mainly relies on photoplethysmography (PPG). Researchers are aiming to use more objective monitoring of PPG to diagnose AMS, mainly focused on heart rate and blood oxygen saturation. While they have been identified as potential early warning indicators of AMS, significant individual variability leads to use them as definitive criteria for AMS diagnosis difficultly. Future research requires enhanced monitoring precision, exploring how individual genetic differences impact hypoxic responses, and developing personalized prevention and treatment strategies in order to provide new insights into high-altitude medicine.

## 1 Introduction

High altitude low pressure and hypoxia can cause a series of physiological and pathological reactions in the human body (Richalet et al., 2024), with changes in heart rate and blood oxygen saturation reflecting the degree of hypoxia. Hypoxia refers to oxygen levels in the body or environment that are below normal levels (Richalet et al., 2024). As altitude increases, air density, atmospheric pressure, and the oxygen pressure in the inspired air all decrease (Richalet et al., 2024). Over 40 million people worldwide reside at altitudes above 2,500 m, and studies on high-altitude populations have primarily focused on Tibetans, Andeans, and Ethiopians. The number of people exposed to high-altitude environments due to work or leisure activities is unknown. With the proliferation of long-haul aviation, billions of passengers experience pressurized cabin conditions corresponding to an altitude of approximately 2,400 m annually. Conversely, military jet fighter pilots are subjected to hypobaric environments equivalent to altitudes ranging from 2,400 to 3,000 m during routine flight training operations. Studies on the physiological and pathological changes in lowlanders exposed to high-altitude hypoxia primarily use low-pressure hypoxia conditions, such as high-altitude environments or low-pressure hypoxia simulation environments; or normal-pressure hypoxia conditions, such as breathing a low-oxygen gas mixture. When oxygen pressure in the inspired air is the same, the results of these studies show no significant physiological differences (Saugy et al., 2016).

In numerous studies conducted in high-altitude hypoxia environments, heart rate and blood oxygen saturation are commonly measured as physiological parameters, with only physiological trends observed. Few studies have explored the underlying molecular mechanisms (such as autonomic nervous system regulation or changes in receptor expression). Additionally, due to the harsh conditions of high-altitude hypoxic environments, heart rate and blood oxygen saturation are typically measured using instruments based on photoplethysmography (PPG). However, the accuracy of these measurements can be affected by factors such as motion artifacts and skin perfusion status, potentially leading to measurement errors. Cerebral tissue oxygen saturation is often used as a supplement to blood oxygen and has been monitored at high altitude in hypoxic environments. In clinical practice, the assessment of acute mountain sickness (AMS) remains largely subjective, relying on the Lake Louise Questionnaire scoring system. Some studies have sought to use readily obtainable heart rate and blood oxygen saturation levels to diagnose AMS.

Acute hypoxia in this review is minutes to hours, subacute hypoxia is weeks, and long-term hypoxia is months or life-long of high-altitude hypoxic exposure. This review focuses on the molecular regulatory mechanisms of cardiovascular function in hypoxic conditions at high altitudes and physiological changes in heart rate and blood oxygen saturation. It also considers the accuracy of commonly used monitoring techniques and their significance in the prevention and treatment of AMS.

## 2 High-altitude hypoxia and oxygen signaling pathways

### 2.1 High-altitude hypoxia and HIF-relative factors regulation

Under hypoxic conditions, prolyl hydroxylase activity is suppressed, preventing the hydroxylation and subsequent degradation of HIF-1α. This stabilization leads to the accumulation of HIF-1α and its dimerization with HIF-1β, thereby activating the transcription of target genes (Zhang et al., 2025). These target genes include erythropoietin, glucose transporter 1, endothelin-1, nuclear factor-κB, nitric oxide synthases, platelet-derived growth factor, and vascular endothelial growth factor (Richalet et al., 2024). The hypoxic stimulus at high altitude reduces blood oxygen saturation, which triggers erythropoietin synthesis and erythropoiesis to enhance oxygen delivery (Alharthi et al., 2023). Erythropoietin exerts pleiotropic effects on the hematopoietic system, notably increasing erythrocyte oxygen-carrying capacity, while also exerting vasoprotective actions through modulation of endothelial and vascular smooth muscle cell function. Glucose transporter one facilitates glucose uptake essential for cardiomyocyte energy metabolism. Endothelin-1 induces vasoconstriction by acting on vascular smooth muscle cells, elevating systemic vascular resistance, and promoting cellular proliferation and migration. Nuclear factor-κB regulates the expression of pro-inflammatory genes, facilitating inflammatory cell infiltration and cytokine release. Nitric oxide synthases catalyzes nitric oxide synthesis, promoting vasodilation and vascular homeostasis. Platelet-derived growth factor stimulates vascular smooth muscle cell proliferation and migration, whereas vascular endothelial growth factor promotes endothelial cell proliferation, migration, differentiation, and neovascularization, contributing to vascular remodeling in hypoxic conditions (Jean-Baptiste et al., 2022). Current data indicates that specific genetic polymorphisms, including EPAS1 and EGLN1, potentially modulate the HIF signaling pathway by attenuating the proteasomal degradation of HIF-α subunits, thereby facilitating high-altitude acclimatization through sustained chemoreceptor responsiveness (Simon et al., 2008). Nonetheless, the enduring impact of genetic adaptation mechanisms on chronic hypoxic conditions remains constrained (Pamenter et al., 2020).

### 2.2 High-altitude hypoxia and energy metabolism

During hypoxic conditions, there is upregulation of glucose transporter proteins and glycolytic enzymes, with the electron transport chain being affected, leading to increased production of mitochondrial superoxide anion (Dunham-Snary et al., 2017). This reactive oxygen species is subsequently dismutated to hydrogen peroxide by mitochondrial superoxide dismutase 2. Concurrently, activation of nicotinamide adenine dinucleotide phosphate oxidase two and xanthine oxidase contributes to reactive oxygen species generation. Cytochrome c oxidase exhibits high oxygen affinity, and HIF-1 activation facilitates subunit switching of cytochrome c oxidase to sustain adenosine triphosphate synthesis and meet cellular energetic demands under hypoxic stress (Lee et al., 2020). Additionally, HIF-1 induces the expression of lactate dehydrogenase A and pyruvate dehydrogenase kinase 1, which inhibit pyruvate entry into the tricarboxylic acid cycle, favor lactate accumulation, and suppress its conversion to acetoacetyl coenzyme A (Gu and June, 2018). Elevated reactive oxygen species levels during hypoxia also increase intracellular Ca^2+^ concentrations, Ca^2+^/calmodulin-dependent protein kinase kinase and adenosine monophosphate-activated protein kinase, with adenosine monophosphate-activated protein kinase activation inhibiting adenosine triphosphate consuming processes (Lee et al., 2020).

## 3 High-altitude hypoxia and population heart rate

### 3.1 Acute hypoxia and heart rate/heart rate variability in lowlanders

Hypoxic stimulation of carotid chemoreceptors, which are sensitive to hypoxaemia, results in activation of the adrenergic nervous system, as indicated by elevated plasma and urinary catecholamine concentrations (Mourot, 2018). Acute hypoxia activates the sympathetic adrenergic nervous system, increasing sympathetic tone and cardiac effects, thereby elevating heart rate (Insalaco et al., 2016). During acute hypoxic episodes, sympathetic nervous system activation results in increased sympathetic tone, leading to tachycardia and reduced heart rate variability (HRV) (Insalaco et al., 2016), thereby impacting cardiorespiratory coupling and autonomic regulation (Su et al., 2024). HRV serves as a robust biomarker for cardiac autonomic function, reflecting the temporal variability between successive R-R intervals and encompassing neurohumoral influences. It provides insights into cardiovascular regulation by autonomic pathways. It is routinely employed in clinical settings to assess cardiovascular risk, autonomic integrity of the sinoatrial node, and to predict circulatory system pathologies (Hou et al., 2023). HRV spectral analysis partitions into four frequency domains: ultra-low frequency, very low frequency, low frequency (LF), and high frequency (HF). LF predominantly correlates with sympathetic nervous system activity, whereas HF is associated with parasympathetic modulation (Task Force of the European Society of Cardiology and the North American Society of Pacing and Electrophysiology ,1996). Reduced HRV has been linked to heightened cardiovascular morbidity and mortality (Su et al., 2024). Hypoxia induces a shortening of the R-R interval, decreases in LF and HF power, and an increased LF/HF ratio, indicative of sympathetic dominance over parasympathetic activity (Yokobori et al., 2023). Cardiomyocytes exhibit heightened sensitivity to fluctuations in oxygen availability, particularly during physical exertion. Exercise in a high-altitude hypobaric environment by people from the lowlands causes a restriction in the utilization of oxygen by cardiomyocytes. Consequently, while heart rate initially elevates during exercise, the maximal exercise heart rate demonstrates a modest reduction (Richalet and Hermand, 2022). Furthermore, heart rate increases at slower running speeds but does not change significantly as running speed increases. Concurrently, peripheral oxygen saturation (SpO_2_) diminishes with increasing running velocity (Li Tee et al., 2023). Research indicates that jet fighter pilots exhibit elevated heart rates as a physiological compensatory mechanism to hypoxic conditions, augmenting cardiac output to sustain tissue oxygenation (Taylor et al., 2025). When deploying a medical helicopter for the evacuation of stable emergency patients capable of autonomous respiration at an altitude of 3,000 m, a reduction in heart rate was observed, potentially attributable to heightened vagal tone. No alterations in respiratory rate were documented, and there was no record of sedative administration (Artero García et al., 2025).

### 3.2 Subacute hypoxia and changes of heart rate in lowlanders

Subacute hypoxia, the sympathetic adrenergic nervous exhibits sustained activation (Simpson et al., 2024), characterized by heightened sympathetic adrenergic nervous activity and elevated synaptic concentrations of noradrenaline and adenosine. However, the downregulation of cardiomyocyte β-adrenergic receptors and adenosine A_1_ receptor, coupled with the upregulation of muscarinic acetylcholine M_2_ receptors, and reduced neuronal reuptake of noradrenaline, results in a decreased heart rate (Richalet, 2016). Stimulatory G-proteins and inhibitory G-proteins mediate the coupling of β-adrenergic receptors to adenylate cyclase, thereby modulating enzymatic activity. These G-proteins may play a critical role in regulating the sympathetic adrenergic nervous during hypoxic conditions. Hypoxia triggers a reduction in stimulatory G-proteins activity, an elevation in inhibitory G-proteins expression, and the suppression of adenylate cyclase activity, culminating in a decreased heart rate (Richalet, 2016). β-arrestin 2 has been implicated in the desensitization and internalization pathways of G protein-coupled receptors (Boussi and Frishman, 2021). The interplay between noradrenaline and β-adrenergic receptors, adenosine and adenosine A_1_ receptor, and acetylcholine M_2_ receptors is crucial for preserving cardiac function. In response to diminished oxygen availability, the body curtails cardiac oxygen consumption to maintain adequate myocardial oxygenation and safeguard the heart from damage linked to elevated energy demands (Richalet and Hermand, 2022). This autoregulatory mechanism of oxygen management shields the heart from ischemic episodes, particularly when the maximal exercise heart rate is reduced compared to acute hypoxia. The decline in maximal exercise heart rate is contingent upon the severity of hypoxia and typically reverses within several days following a return to sea level (Mourot, 2018). The most compelling physiological rationale for the reduction in maximal exercise heart rate during subacute hypoxia, in contrast to acute hypoxia, is the downregulation of β-adrenergic receptors (Richalet et al., 2024). Nevertheless, it has also been demonstrated that the decrease in maximal exercise heart rate stems from the uncoupling of β-adrenergic receptors from G-proteins or second messenger phosphorylation, rather than a straightforward downregulation of β-adrenergic receptors (Mourot, 2018). A study involving male adolescents from lowlands, monitored for HRV changes in a high-altitude environment at 3,680 m above sea level over 4 weeks, performing 20-min exercises at low, medium, and high intensities, revealed that both moderate and high-intensity exercise effectively mitigated hypoxia-induced HRV reduction, with high-intensity exercise showing a more pronounced effect. Researchers attributed this to the study’s use of relative intensity, determined by the percentage of individual reserve heart rate, and the short intervention period of 4 weeks with 20-min exercise sessions, which may have influenced the impact of aerobic training on HRV (Su et al., 2024).

### 3.3 Long-term hypoxia and changes of heart rate in lowlanders

Long-term exposure to high-altitude hypoxia affects the regulation of autonomic nervous system activity, shifting from predominantly sympathetic adrenergic nervous activity to enhanced parasympathetic activity (Taralov et al., 2018), which maintains overall internal homeostasis through the body’s autoregulatory mechanisms (Richalet, 2016). Long-term hypoxia induces increased parasympathetic activity, which may be associated with reduced heart rate (Mourot, 2018). During extended hypoxia, the resting heart rate decreases relative to subacute hypoxia, ultimately reverting (return) to baseline (Richalet, 2010). Long-term hypoxia diminishes human exercise capacity, reduces maximal oxygen uptake (Mourot, 2018), and significantly lowers maximal exercise heart rate compared to subacute hypoxia. In a high-altitude cycling study, researchers found that daily HRV monitoring during a 5-month training period could predict changes in exercise capacity; however, whether this holds during competition remains uncertain. Researchers suggest that the overload and potential performance decline in athletes during competition may hinder the accurate prediction of exercise capacity via HRV monitoring (Bourdillon et al., 2023). In athletes utilizing hyperoxic recovery post-hypoxic exercise at altitude, short-term cellular energy supply improved, but athletic performance did not. Prolonged hyperoxic recovery may attenuate hypoxic exercise-induced adaptive responses, while the increased production of reactive oxygen species during hyperoxic recovery elevates the risk of hypoxic injury (Burtscher et al., 2024).

### 3.4 Heart rate characteristics of highlanders under high-altitude hypoxia

Andean at elevations exceeding 3,000 m exhibit a mean heart rate of approximately 71 beats per minute (Eichstaedt et al., 2015). A comparative analysis revealed that Andeans dwelling in Peru at altitudes above 3,000 m for a decade demonstrated elevated heart rates relative to lowland populations residing in the Lima region of Peru for an equivalent duration (Caffrey et al., 2014). Furthermore, at elevations exceeding 4,300 m, the Tibetan hereditary population (*n* = 730) exhibited a lower heart rate compared to the Han Chinese population (*n* = 30) (Xu et al., 2018).

## 4 High-altitude hypoxia and blood/cerebral oxygen saturation in the population

### 4.1 High-altitude hypoxia and blood oxygen saturation in lowlanders

Upon ascent to 5,380 m, lowlanders exhibited alterations in pulse oximetry readings. Acute hypoxic exposure reduced alveolar oxygen partial pressure, impairing oxygen exchange and leading to decreased blood oxygen saturation, with nadir values observed on the initial day. Prolonged subacute exposure facilitated a gradual recovery of blood oxygen saturation, albeit remaining below baseline sea-level values (Yan et al., 2023). Long-term high-altitude hypoxia prevented complete restoration of baseline blood oxygen saturation, despite erythrocytosis and increased blood viscosity (Yan et al., 2023). After 2 years of high-altitude exposure, adult Han Chinese males demonstrated SpO_2_ exceeding 90% (Liang et al., 2024). Following 21 days of high-altitude exposure, lung volumes were reduced, accompanied by vasodilation, decreased pulmonary artery pressure, and elevated SpO_2_ (Song et al., 2023). The observed increase in SpO_2_ may represent an adaptive response, potentially linked to elevated lactate levels (Hutcheon et al., 2023).

High-altitude hypoxic sleep studies revealed a slight reduction in mean SpO_2_ during cyclic breathing, a more significant decrease during acute hypoxia, and an increase during subacute hypoxia (Insalaco et al., 2012). In healthy preterm adults, the resting brain tissue saturation index was higher than in term adults following acute high-altitude hypoxia. The resting brain tissue saturation index is higher in healthy premature adults than in full-term adults after acute high-altitude hypoxic exposure. Healthy premature adults are also better able to maintain brain tissue saturation index and pulse oxygen saturation during high-altitude exercise (Narang et al., 2024). Nocturnal sleep in the preterm group exhibited diminished SpO_2_ recovery and more frequent pulse oximetry desaturations, with a mean SpO_2_ ≤ 77.3% predictive of AMS (Narang et al., 2025). Patients with pulmonary hypertension and chronic thromboembolic pulmonary hypertension exhibit lower SpO_2_ levels at high altitude, both diurnally and nocturnally, compared to healthy subjects, with a more significant increase in the LF/HF ratio at night, indicating exacerbated autonomic dysregulation (Herzig et al., 2024). Continuous positive airway pressure ventilation improves oxygen saturation at rest and during exercise at high altitudes, thereby correcting hypoxaemia. As a portable device, continuous positive airway pressure can be used to prevent and treat altitude-related illnesses, as well as enhance safety in high-altitude rescue scenarios (Strickland et al., 2024).

Full-term newborns at sea level exhibited pulse oximetry of 50% at birth, exceeding 90% within 10 min and 95% within 15 min, attributed to lung activation, decreased pulmonary arterial pressure, and the contraction and closure of the arterial duct caused by an increase in oxygen partial pressure. As altitude increases, pulse oxy-saturation values in full-term newborns show a negative correlation with altitude. Within 30–120 min after birth, these values were measured as 94% in Xishuangbanna (847 m), 92% in Kunming (1,983 m), 89% in Shangri-La (3,509 m), and 83% in Yushu (4,360 m) (Li et al., 2023).

Research indicates that during simulated high-altitude training at 7,000 m within a controlled hypobaric chamber, with pilots donning oxygen masks, there is a progressive decline in arterial oxygen saturation as simulated altitude increases. Specifically, after approximately 200 s of hypoxic exposure, the mean pulse oximetry readings fall below 60%. Upon removal of the oxygen mask, the arterial oxygen saturation exhibits an exponential decline, reaching critically low levels (Nakdimon et al., 2025).

### 4.2 High-altitude hypoxia and blood oxygen saturation in highlanders

In high-altitude populations, the Andean population residing at elevations exceeding 3,000 m exhibits an average SpO_2_ of approximately 87% (Eichstaedt et al., 2015), while Tibetans at 3,000–4,000 m demonstrate a blood oxygen saturation of roughly 89% and a lower blood oxygen saturation compared to Han Chinese migrants at equivalent altitudes (He et al., 2023). Ethiopians inhabiting the East African plateau display hemoglobin and SpO_2_ levels comparable to those of lowland populations, with limited research available on this demographic, suggesting a potential superior adaptation to the hypobaric conditions of the plateau (Richalet et al., 2024).

### 4.3 High-altitude hypoxia and cerebral blood flow, and cerebral tissue oxygen saturation monitoring at sea level

**FIGURE 1:**
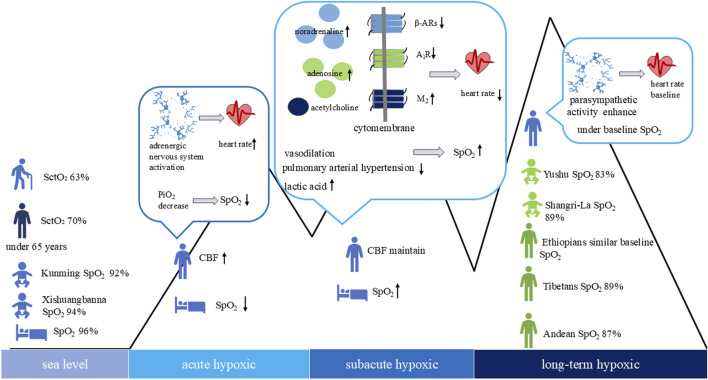
High-altitude hypoxia and sea level with heart rate, blood/brain or cerebral tissue oxygen saturation in the population cerebral tissue oxygen saturation (SctO_2_), cerebral blood flow (CBF), peripheral oxygen saturation (SpO_2_).

Cerebral tissue oxygen saturation comprehensively reflects the oxygenation status of hemoglobin in the brain microcirculation (small arteries, capillaries, and veins) and is currently used primarily as a supplementary indicator for clinical blood oxygen monitoring. However, research on cerebral hemodynamics and brain tissue oxygen saturation in high-altitude hypoxia environments remains scarce. In response to acute high-altitude hypoxia, cerebral blood flow elevates to maintain cerebral oxygen delivery, counteracting the reduction in arterial oxygen content, with normalization occurring within 48 h (Turner et al., 2024). At 8,000 m, cerebral blood flow autoregulation is compromised, leading to cognitive impairment (Richalet, 2010). Cerebral tissue oxygen saturation measured via cerebral oximetry at sea level reveals an average of 70% in individuals under 65 years, decreasing to approximately 63% in older populations (Lian et al., 2020). High-altitude hypoxia and sea level with blood/brain or cerebral tissue oxygen saturation in the population, as shown in Figure 1.

## 5 Monitoring devices commonly used for heart rate and blood oxygen saturation, and their potential application for AMS diagnosis under high-altitude hypoxia

### 5.1 Monitoring devices commonly used for heart rate and blood oxygen saturation in high-altitude hypoxic environment

The internationally established gold standard for cardiac rhythm assessment is the electrocardiography (ECG) (Quigley et al., 2024), which provides measurements of heart rate and HRV. The benchmark for oxygen saturation assessment is arterial blood gas analysis (Abraham et al., 2023), typically involving arterial puncture of the radial artery to determine arterial oxygen saturation (SaO_2_) and partial pressure of oxygen. Commonly employed ECG modalities include 12-lead ECG and ambulatory Holter monitoring; the 12-lead ECG offers superior measurement accuracy but is limited by high cost, lack of portability, and the need for trained personnel (Quigley et al., 2024). In recent high-altitude physiology research, few studies have utilized ECG for heart rate monitoring, with unspecified device types. Similarly, arterial blood gas analysis for oxygen saturation is infrequently applied due to the procedural risks and logistical challenges of biological sample collection in extreme environments at high altitude. Additionally, arterial blood gas analysis sampling is invasive, operator-dependent, and necessitates strict aseptic techniques (Joyce et al., 2024).

Advancements in biomedical engineering have facilitated the development of a non-invasive, portable PPG system based on optical sensing technology. This modality primarily relies on the measurement of volumetric pulse wave signals derived from light-tissue interactions, achieved through irradiation of the skin with specific wavelengths and detection via infrared emitters coupled with phototransistors (Quigley et al., 2024). Common light sources include infrared, red, and green light emitting diodes (LED), which detect reflected or transmitted light signals for further analysis (Wójcikowski and Pankiewicz, 2020). Infrared LED offers superior tissue penetration, enabling the assessment of deep vascular blood flow, although it cannot differentiate between arterial and venous blood compartments and is vulnerable to ambient light interference and motion artifacts. Green LED, characterized by a shorter wavelength, penetrates superficial tissues more effectively, providing enhanced accuracy in quantifying oxygenation levels of oxyhemoglobin and deoxyhemoglobin in vascular-rich regions, though it remains sensitive to skin pigmentation, perspiration, and other surface conditions. Red LED exhibits deeper tissue penetration with reduced dependence on skin tone, making it suitable for low-perfusion states and multi-parameter monitoring; however, it presents limitations in measurement precision and is prone to ambient light interference and higher energy consumption. Hybrid systems combining infrared and red wavelengths have demonstrated improved accuracy in SpO_2_ measurement (Kavsaoğlu et al., 2015; Lyra and Paul, 2019). Compared to ECG and arterial blood gas analysis, PPG provides non-invasive estimation of heart rate and oxygen saturation. Nonetheless, the technology’s susceptibility to variations in skin pigmentation and surface moisture can introduce measurement bias (Al-Halawani et al., 2023).

ISO 80601-2-61 delineates the international standard for medical pulse oximetry utilizing PPG technology. The SpO_2_ measurement range is 70%–100% (with a transmission variance of ≤3.0% and an ear clip/reflectance variance of ≤3.5%), and the heart rate measurement range is 25–250 beats per minute. However, when the SpO_2_ value is below 70%, the margin of error increases when using this method. Measurement accuracy diminishes when SpO_2_ values fall below 70% (Leppänen et al., 2022; Dünnwald et al., 2021). PPG sensors are deployable on various anatomical sites, including fingertips, wrists, earlobes, forehead, torso, ankles, and nasal region, with wrist placement being predominant. The wrist offers advantages such as higher bone mineral density, which enhances reflected light signal acquisition (Wójcikowski and Pankiewicz, 2020), and the modality’s cost-effectiveness, portability, and user-friendliness (Castaneda et al., 2018). Nonetheless, daily wrist movements can compromise sensor-skin contact, leading to measurement artifacts. Additionally, the signal strength at the wrist is weaker than that of the peak amplitude signals at other parts of the body, such as the fingers (Wójcikowski and Pankiewicz, 2020). Discrepancies between SaO_2_ obtained via arterial blood gas analysis and SpO_2_ from pulse oximetry have been documented, particularly in hypobaric, hypoxic environments, exhibiting considerable deviation (Ottestad et al., 2018).

Portable PPG devices have become integral to high-altitude medicine research. Compared to ECG, the gold standard for heart rate monitoring, and arterial blood gas analysis for oximetry, PPG technology offers advantages such as enhanced convenience, rapid data acquisition, and non-invasive assessment. PPG-based monitoring systems have been employed extensively in high-altitude physiology studies (Kitsiripant et al., 2017; Gudelunas et al., 2024; Hiscock et al., 2015; Nazari et al., 2019; Nazari and MacDermid, 2020; Littell et al., 2022), as summarized in Table 1. Additionally, cerebral tissue oxygen saturation shows a strong correlation with SpO_2_ at levels ≥70%, suggesting cerebral tissue oxygen saturation as a valuable adjunct in both clinical management and hypoxia training (Ottestad et al., 2018). Altered respiratory function and reduced blood oxygen saturation in high-altitude hypoxic environments result in relatively higher SpO_2_ compared to SaO_2_. Consequently, pulse oximetry is insufficient for clinical decision-making (Prosperi et al., 2023). As a contact-based PPG device signal integrity can be compromised by suboptimal skin contact during extended monitoring periods, influenced by factors such as perspiration, necessitating individual attachment during data acquisition. To reduce motion artefacts that affect measurement accuracy, several non-contact devices have emerged, such as imaging photoplethysmography (Yizhuo et al., 2025) and remote photoplethysmography (Rao et al., 2025).

**TABLE 1:** Comparison between different instruments for monitoring heart rate and blood oxygen saturation.

Equipment	Grade	Heart rate accuracy	SpO_2_ accuracy	References
Masimo Radical-7®	Medical grade	Higher accuracy (0.5 breaths/min deviation from standard method)	High accuracy of SpO_2_ monitoring (−0.4 per cent deviation)	Gudelunas et al. (2024), Hiscock et al. (2015), Kitsiripant et al. (2017)
Nellcor PM1000N	Medical grade	PPG-based, accuracy affected by motion	SpO_2_ deviation of −0.4% (95% CI: −0.5% to −0.3%) compared to Masimo Radical-7®	Kitsiripant et al. (2017)
Zephyr Bioharness	Medical grade	High accuracy at rest (ICC 0.94–0.97), poor accuracy during exercise (ICC 0.31–0.99, recovery ICC 0.44–0.98)	-	Nazari and MacDermid (2020), Nazari et al. (2019)
Nellcor N-595	Medical grade	-	Increased bias in low perfusion and dark skin conditions; misdiagnosis rates of 8.2% and 21.1% in medium and dark skin populations	Gudelunas et al. (2024)
Masimo Radical 7	Medical grade	-	Increased bias in low perfusion and dark skin conditions; misdiagnosis rate of 30.2% in dark skin population	Gudelunas et al. (2024)
Fitbit Charge HR	Consumer grade	High accuracy at rest (ICC 0.92–0.97), poor accuracy during exercise (ICC 0.45–0.99, recovery ICC 0.45–0.98)	-	Nazari et al. (2019)
Apple Watch Series 6	Consumer grade	High accuracy of heart rate monitoring (RR interval error 42.5 ± 44.2 ms)	The mean difference compared to standard pulse oximetry was 2.0% ± 2.6%, but was greater in hypoxaemia (SpO_2_ < 90%)	Littell et al. (2022)

SpO_2_, peripheral oxygen saturation; PPG, photoplethysmography; ICC, interclass correlation coefficient; CI, confidence intervals.

### 5.2 Limitations of heart rate and blood oxygen saturation in the diagnosis of AMS

As increasing numbers of individuals engage in high-altitude travel or occupational activities, the incidence of AMS has been observed to correlate positively with elevation, as quantified by the Lake Louise Questionnaire scoring system (Berger et al., 2023). AMS represents a nonspecific, functional hypoxic response to rapid ascent, presenting with symptoms such as cephalalgia, nausea, and fatigue. Although typically non-fatal, it may escalate to severe high-altitude illnesses, including high-altitude pulmonary edema and high-altitude cerebral edema (Berger et al., 2020). The Lake Louise Questionnaire is widely employed in both research and clinical practice to evaluate AMS severity, though its assessments are inherently subjective (Luks et al., 2024). Given the practicality of measuring heart rate and blood oxygen saturation in high-altitude environments, research has explored utilizing blood oxygen saturation, HRV and heart rate as supplementary biomarkers for AMS diagnosis and severity assessment (Lun et al., 2020; Koehle et al., 2010; Zeng et al., 2024; Sutherland et al., 2017; Bian et al., 2015; Li et al., 2015).

Studies indicate that individuals have a relatively low risk of AMS when their blood oxygen saturation levels are high, and monitoring changes in blood oxygen saturation at the beginning of an individual’s journey to high altitude can be beneficial in identifying their potential AMS risk (Lun et al., 2020). For instance, individuals with SpO_2_ ≥ 86% at altitudes above 4,380 m exhibit a low incidence of AMS (Koehle et al., 2010). In a research conducted by Zeng et al., the utilization of smartwatch-based SpO_2_ monitoring at elevations of 4,000 m or higher demonstrated high reliability and precision in the diagnosis and prognostication of AMS (Zeng et al., 2024). However, Sutherland et al. found that HRV assessment under normoxic conditions yielded superior diagnostic accuracy for AMS prediction compared to HRV measurements in hypoxic environments, and was more precise than SpO_2_ monitoring (Sutherland et al., 2017). Additionally, Bian et al. reported that individuals with AMS exhibited a statistically significant elevation in heart rate compared to those without AMS (Bian et al., 2015). Elevated heart rate has also been associated with AMS, with increases exceeding 25 beats per minute after acute exposure to hypoxia or more than 15 beats per minute following pre-acclimatization, indicating heightened risk (Li et al., 2015). The primary intervention for AMS is descent to lower altitudes; however, supplemental oxygen therapy can serve as an alternative, aiming to maintain SpO_2_ above 90% to mitigate hypoxic stress and alleviate symptoms (Luks et al., 2024). Further validation of wearable devices, such as smartwatches, in diverse high-altitude environments is necessary to compare their performance with medical-grade pulse oximetry (Zeng et al., 2024).

## 6 Conclusion

High-altitude hypoxia regulates the cardiovascular system by stabilizing hypoxia-inducible factor and controlling the expression of downstream target genes while inhibiting the electron transport chain and the tricarboxylic acid cycle, thereby increasing glycolysis and reducing cellular energy consumption. Under acute hypoxia, blood oxygen saturation decreases, and the adrenergic nervous system is activated, resulting in an accelerated heart rate. Under subacute hypoxia blood oxygen saturation may rebound but will remain below baseline values at sea level, meanwhile heart rate is gradually reduced by the downregulation of the adrenergic system and the expression levels of β-adrenergic receptors and adenosine A_1_ receptor, as well as the upregulation of muscarinic M_2_ receptors. Under long-term hypoxia heart rate returns to baseline values, but blood oxygen saturation remains below baseline. High-altitude hypoxia causes a decrease in heart rate variability, exercise in a high-altitude hypoxic environment is beneficial in improving heart rate variability. Heart rates were lower in the Tibetan compared to the Han Chinese at equivalent altitudes. Conversely, heart rates were elevated in the Andean relative to the Peruvian lowlanders. Moreover, Tibetan and Andean populations exhibited lower peripheral oxygen saturation compared to the lowlanders, whereas the Ethiopian population displayed comparable peripheral oxygen saturation. Cerebral blood flow, as a complementary indicator to blood oxygen monitoring, has been studied to monitor cerebral tissue oxygen saturation and its changes in high-altitude hypoxic environments. In the harsh environment of high altitude and low oxygen, photoplethysmography -based devices are mainly used in practical applications, limited to the availability of gold standards for heart rate and oxygen saturation monitoring (e.g., electrocardiography and arterial blood gas analysis). However, photoplethysmography monitoring is susceptible to interference from motion artifacts, sweat, and other factors. Multimodal monitoring techniques are expected to be utilized to improve monitoring accuracy. For individuals who are not acclimatized to high altitudes, blood oxygen saturation below 86% and an increase in heart rate of 25 bpm are key early warning signs of acute mountain sickness. However, these thresholds cannot be used as diagnostic criteria for acute mountain sickness due to significant differences between individuals. Future research should focus on refining monitoring techniques and elucidating the impact of individual genetic differences on hypoxic responses. Personalized high-altitude medicine necessitates a collaborative approach integrating physiology, engineering, and clinical practice.
